# Food and Nutrient Intake in Children and Adolescents with or without Overweight/Obesity

**DOI:** 10.3390/nu15204450

**Published:** 2023-10-20

**Authors:** Yan Zou, Lichun Huang, Dong Zhao, Mengjie He, Dan Han, Danting Su, Ronghua Zhang

**Affiliations:** Zhejiang Provincial Center for Disease Control and Prevention, Hangzhou 310051, China; zouyan0573@163.com (Y.Z.);

**Keywords:** nutrient, food, children, China, overweight, obesity

## Abstract

Objective: To explore the food and nutrient characteristics of children and adolescents with or without overweight/obesity and to provide scientific basis for the development of prevention strategies on overweight/obesity. Methods: This study included children and adolescents aged 6–17 years who participated in provincial nutrition surveillance which included 90 counties (cities and districts) in Zhejiang Province with a provincial representative. Food consumption was assessed using three consecutive days of 24 h dietary recall, and nutrient intake was calculated using dietary recall in conjunction with the China Food Composition Table. Overweight/obesity was also investigated. Associations were evaluated using ordinal regression models. Results: The analysis included a total of 1827 children and adolescents. The overweight prevalence was 14.0% and the obesity prevalence was 10.1%. Children and adolescents with overweight/obesity were observed to have a higher intake of carbohydrates, iron, sodium, potassium, and magnesium (F = 3.464, 5.232, 5.619, 3.469, 3.934, *p* < 0.05), as well as having a higher intake of the food group of cereals, tubers and beans, snacks, and salt (F = 7.348, 6.797, 3.413, *p* < 0.05) compared to children and adolescents without overweight/obesity. After adjusting for potential confounders via ordinal regression models, children and adolescents with overweight/obesity were observed to have a higher intake of carbohydrates (Wald χ^2^ = 4.325, *p* < 0.05). There were significant differences concerning the daily energy provided by snacks, the daily carbohydrate intake provided by snacks, the daily sodium intake provided by snacks, and the proportion of snacks to the food group of cereals, tubers, and beans (F = 8.305 6.316, 13.955, 3.692, *p* < 0.05). Conclusion: Children and adolescents with overweight/obesity have presented a high consumption of carbohydrates, which is associated with the food group of cereals, tubers, and beans. Snacks are the main factor leading to the high intake of the food group of cereals, tubers, and beans among children and adolescents with overweight/obesity.

## 1. Introduction

Good nutrition is essential for optimal growth and development in children and adolescents. Poor childhood nutrition increases the risk of non-communicable diseases [1]. The association between higher BMI and chronic diseases is well established, and epidemiological evidence shows that obesity is an independent risk factor for many diseases, including type 2 diabetes, cardiovascular disease, non-alcoholic fatty liver disease, sleep apnea, hypertension, stroke, and several cancers [2,3]. For children and adolescents, obesity can also have a negative impact on the cognitive function, which may lead to a decrease in academic performance [4]. The global obesity rate in children is rapidly increasing, with the obesity rate in children increasing more than 8 fold in the past 40 years [5], and it is estimated that the obesity rate of children and adolescents aged 5–19 will reach 40 million and 254 million by 2030 [6]. Obesity is caused by a set of complex interactions between the environment, genetic predisposition, and human behavior [7]. Presumed risk factors that can be modified in children and adolescents without professional intervention include diet and body weight [8]. Diet is an important factor. Dietary intake plays an important role in the health of children and adolescents. The early detection of dietary risk factors and the implementation of targeted prevention and early identification are the most cost-effective means for overweight/obesity prevention and control. Starting from identifying the risk factors for primary disease prevention, it is crucial to focus on precise interventions in the first half of the period from healthy to the onset. At present, the sources of nutrient intake in overweight/obese children and adolescents, the changes in food proportion in food categories caused by economic and social development, and the key food related to overweight/obesity in food categories urgently need to be compared and analyzed in order to identify the core intervention links for controlling overweight and obesity and dietary nutrition. Our study determined the food and nutrient intake of children and adolescents and explored the disparities of the food and nutrient intake between children and adolescents with or without overweight/obesity. Our findings will inform potential intervention targets and strategies for improving the quality of dietary intake and reduce the prevalence of overweight/obesity.

## 2. Methods

### 2.1. Study Design and Participants

This study included children and adolescents aged 6–17 years who participated in provincial nutrition surveillance, which included 90 counties (cities and districts) in Zhejiang Province with a provincial representative. The field investigation and physical examinations were conducted in 2022. The sample size is calculated using the formula N = $\frac{{deff\;u}^{2}P(1-P)}{d^{2}}$. “deff” is the design efficiency, and in the sample size calculation of this study, the “deff” value is set to 1.5. “d” is the relative error, which is controlled at 15% in this study. The confidence level is set at 95% (bilateral), so “u” is 1.96. “P” is the obesity rate of the previous year. The expected number of respondents is 1600, while the actual number of respondents is 1867. Children and adolescents were divided into age groups by age 6–8, 9–11, 12–14, and 15–17.

### 2.2. Anthropometric Measurements

Anthropometric measurements were taken by trained health workers. Height was measured without shoes to the nearest 0.2 cm using a portable stadiometer (TZG; Wuxi Weighing Instrument Factory Co., Ltd., Wuxi, China), and weight was measured without shoes and in light clothing to the nearest 0.1 kg on a calibrated beam scale (G&G TC-200k; Shanghai Taizhiheng Electronic Weighing Instrument Co., Ltd., Shanghai, China). Body mass index (BMI) was calculated as weight (kg)/height (m)^2^. We defined being overweight and obese using the sex- and age-specific BMI (kg/m^2^) cut-off points. Overweight and obese children were screened (Screening for Overweight and Obesity Among School-Age Children and Adolescents; issued by the State Health and Family Planning Commission of the People’s Republic of China. WS/T 586-2018 [9]) and the prevalences of being overweight and obese were calculated.

### 2.3. Dietary Intake

During home visits, dietary data on breakfast, lunch, dinner, and extra meals or snacks were collected via interviews. A dietary survey was conducted using the Chinese Resident Nutrition and Health Survey (CNHS) questionnaire to ensure the quality of dietary recalls. The 24 h retrospective method was used to conduct a 3-day personal food intake survey of all family members, to record the amount of food consumed (including meals outside), and to calculate the nutrient intake per person. The whole family was interviewed, but the food and nutrient intake of children and adolescents was analyzed in this study. The parents recorded their child’s dietary intake. The amounts of food consumed by the children were estimated using a standardized bowl and food atlas. We calculated the nutrient intake using the China Food Composition Table published in 2002 [10]. We combined our food intake data with the food nutrient content in the China Food Composition Table to calculate the nutrient intake of each child. The quantities of salt and edible oil were extra from that which were in the other forms of food groups. We verified the data before analysis and excluded implausible data points, such as an energy intake <500 kcal or >5000 kcal per day.

### 2.4. Ethical Considerations

The study was conducted in accordance with the Declaration of Helsinki and approved by the Ethics Committee of Zhejiang Provincial Center for Disease Control and Prevention (approval number: AF/SC-06/01.0, date of approval: 28 January 2022). The research protocols were carefully explained to the parents/guardians of the primary school children, and all participants and parents/guardians of the primary school children provided written informed consent.

### 2.5. Statistical Analysis

Data processing and statistical analyses were performed using SAS 9.2 software (SAS Institute, Cary, NC, USA). The continuous variables with normal distributions were presented as mean ± standard deviations (SD). Differences in the daily food and dietary nutrient intake among children and adolescents with or without overweight/obesity were assessed using ANOVA. Associations were evaluated using ordinal regression models. In univariate analysis, variables with a *p*-value less than 0.1 were included in the ordinal regression, and age group and gender were also included in the ordinal regression. All tests were two-sided, and the level of significance was set at *p* < 0.05.

## 3. Results

### 3.1. Study Population

The study included 1827 children and adolescents and the response rate is 97.8%. The overweight prevalence was 14.0% and the obesity prevalence was 10.1%. The overweight prevalence in children aged 6–8, 9–11,12–14, and 15–17 was 11.5%, 14.5%, 15.8%, and 14.6%, respectively. The obesity prevalence in children aged 6–8, 9–11,12–14, and 15–17 was 12.1%, 11.0%, 9.0%, and 7.7%, respectively. There were no significant differences among children aged 6–8, 9–11,12–14, and 15–17 with or without overweight/obesity (χ^2^ = 8.849, *p* = 0.182).

### 3.2. Comparison of Nutrient Intake among Children and Adolescents with or without Overweight/Obesity

There were significant difference of the daily intake of carbohydrates, iron, sodium, potassium, and magnesium among children and adolescents with or without overweight/obesity (F = 3.464, 5.232, 5.619, 3.469, 3.934, *p* < 0.05) (Table 1). Although the daily intake of protein was higher in children and adolescents with overweight/obesity, it did not reach a statistical difference (F = 2.565, *p* = 0.078).

### 3.3. Comparison of Food Intake among Children and Adolescents with or without Overweight/Obesity

There were significant differences in the intake of the food group of cereals, tubers and beans, snacks, and salt among children and adolescents with or without overweight/obesity (F = 7.348, 6.797, 3.413, *p* < 0.05) (Table 2). Although the daily intake of vegetables was higher in children and adolescents with overweight/obesity, it did not reach a statistical difference (F = 2.640, *p* = 0.072).

Ordinal regression shows that the food group of the cereals, tubers, and beans intake was associated with overweight/obesity of children and adolescents (χ^2^ =2.804, *p* = 0.038) (Table 3).

There were significant differences of the daily energy provided by snacks, the daily carbohydrate intake provided by snacks, the daily sodium intake provided by snacks, and the proportion of snacks to the food group of cereals, tubers, and beans (F = 8.305, 6.316, 13.955, 3.692, *p* < 0.05) (Table 4).

## 4. Discussion

In the past decade, the rate of being overweight and obese in developed countries has approached or reached a peak. According to a 2016 report by the World Health Organization, the rate of being overweight among adolescents aged 10–19 years in the United States, Canada, New Zealand, Australia, the United Kingdom, Spain, Israel, and other developed countries exceeds 30%, and the rate of obesity exceeds 10% [11]. Increasing BMI is a global health concern affecting high- and low-income countries [3]. The high rates of being overweight and obese among children and adolescents represent a global public health problem that needs to be solved urgently. Children with overweight/obesity have a significantly increased risk of many health problems in the future, including adult obesity, type 2 diabetes, and heart disease. Despite this relentless growth, common sense methods aimed at prevention and treatment have not been able to solve this problem. Current prevention methods face significant challenges.

Based on present cross-sectional observation, overweight/obesity children and adolescents consumed significantly more dietary carbohydrates, which is associated with the food group of cereals, tubers, and beans than their counterparts without overweight/obesity. Snacks are the main factor leading to the high intake of the food group of cereals, tubers, and beans among children and adolescents with overweight/obesity. The results of this study identify high-risk factors for preventable dietary nutrition, which is of great significance in reducing the occurrence of overweight and obesity from the source.

### 4.1. Nutrient Intake

Energy balance and macronutrient balance are the cornerstone of any obesity theory that must be established. Obesity only occurs when the energy intake is consistently higher than the energy expenditure. However, the macronutrients in the diet can also affect energy balance. The overall macronutrients in the diet, rather than just the calorie intake, may affect weight. However, the relationship between the energy of carbohydrates, proteins, and fats and obesity is not yet clear. Frank M reported that reduced-calorie diets result in clinically meaningful weight loss regardless of which macronutrients they emphasize [12]. Some studies have shown that a high unsaturated fat diet, a low-fat diet, or a high-carbohydrate vegetarian diet can lead to weight loss [13,14]. A study carried out in Central Jakarta found that obese women had significantly higher intakes of energy, carbohydrates, and fat compared with normal-weight women; however, their protein intake and energy density did not differ significantly [15]. The protein leverage hypothesis proposes that a preference for protein in combination with a decrease in the ratio of protein to carbohydrates and fat in the diet is related to an excess energy intake and the risk of obesity [16]. The relevance of macronutrient ratios or of the combinations of carbohydrate and protein sources for body composition remains to be elucidated. Diet programs—some focusing on carbohydrate reduction and others on fat reduction—have been promoted widely by the media and have generated intense debates about their relative merit [17]. The introduction of carbohydrate-rich foods can counteract adverse stimuli, stimulate the release of serotonin, and thus have a positive impact on emotions [18]. Snacking habits have also been suggested as a potential cause, contributing to the development of overweight and obesity among children. In this study, we found the difference of the daily intake of carbohydrates among children and adolescents with or without overweight/obesity. The nutritional composition of ultra-processed snacks is generally worse than that of unprocessed food. Ultra-processed foods contain more carbohydrates than non-ultra-processed foods, most of which come from low-quality carbohydrates, which is reflected in the high proportion of calories added with sugar and the low levels of dietary fiber and protein. In this study, although the daily intake of protein was higher in children and adolescents with overweight/obesity, it did not reach a statistical difference. This finding was consistent with the literature that suggests that, a low carbohydrate diet is more effective in weight loss than a low fat diet [19]. The optimal intake of specific nutrients is essential for maintaining immune components within the normal range of activity to prevent and overcome infection. The European Food Safety Agency identified six vitamins (D, A, C, folic acid, B6, and B12) and four minerals (zinc, iron, copper, and selenium) as essential for normal immune system function [20]. The intakes of excess sodium and insufficient potassium are two major contributors of heart diseases and stroke development [21]. China has formulated a salt reduction plan. In this study, we found that children and adolescents with overweight/obesity have a higher sodium and potassium intake than those without overweight/obesity. We propose two reasons to explain these results. Firstly, the proportion of food types in the carbohydrate intake of overweight/obesity children and adolescents may affect the sodium intake, such as an increased sodium intake due to the intake of snacks. A study about snacks and overall diet quality found that the sodium component scores were positively associated with snacking frequency [22]. Another study about the dynamics of the Chinese diet found that the changes in cooking and eating styles include a decrease in the proportion of food steamed, baked, or boiled, and an increase in snacking and eating away from home [23]. Secondly, overweight/obesity children and adolescents also consume a higher amount of foods rich in potassium, such as vegetables. Although guidance for obese children and adolescents should reduce their salt intake, it may not be entirely clear which foods to avoid. Therefore, the knowledge of pre-packaged food labeling urgently needs to be popularized [24]. A study on dietary minerals in lactating Women in China found that considering the dietary sources of minerals, 27.3% of iron was from animal-based food [25]. In this study, we found that children and adolescents with overweight/obesity have a higher iron and magnesium intake than those without overweight/obesity. The reasons to explain these results is that overweight/obesity children and adolescents consume a higher amount of foods rich in iron, such as meat, and foods in magnesium, such as vegetables and coarse cereals.

### 4.2. Food Intake

The traditional Chinese diet includes large amounts of cereals and vegetables and small amounts of animal-source foods [23]. But, with the acceleration of modern life’s pace, people’s demand for convenient food has become increasingly urgent. Therefore, people have more opportunities to access a range of packaged foods, many of which are snacks, and these foods are likely to become a major component of their diet in the next decade [26]. In this study, we found that children and adolescents with overweight/obesity have a higher daily intake of the food group of cereals, tubers, and beans. We also found that the daily energy provided by snacks, the daily carbohydrates intake provided by snacks, the daily sodium intake provided by snacks, and the proportion of snacks to the food group of cereals, tubers, and beans was higher in children and adolescents with overweight/obesity. Multivariate analysis considering age and gender factors found that that the food group of the cereals, tubers, and beans intake was associated with overweight/obesity of children and adolescents. Further analysis revealed that children and adolescents with overweight/obesity had higher levels of energy, carbohydrate intake, and sodium provided by daily consumed snacks than their counterparts without overweight/obesity. These findings were consistent with the literature about the dynamics of the Chinese diet [23]. Meanwhile, studies of dietary patterns suggested that the high intakes of rice, vegetables, and pork represent a traditional southern dietary pattern [27]. In this study, we found that the daily intake of vegetables was higher in children and adolescents with overweight/obesity, but it did not reach a statistical difference, suggesting that the high intakes of the food group of cereals, tubers, and beans may play a major role in the difference between children and adolescents with or without overweight/obesity from another perspective. Snack foods-typically high in salt, sugar, fat, and/or energy are likely important to the obesity epidemic [28]. Pre-packaged snacks are one of the main causes of overweight and obesity in children and adolescents [29].

There are reports that eating snacks can help individuals achieve the recommended intake of fruits, dairy products, vitamins, minerals, and fiber and can also help individuals avoid digestive and metabolic overload caused by eating too few or too many meals [30], while there are also reports that snacks can lead to overweight and obesity [31]. Strategic selection of healthy snack options can improve diet quality. But, in southwest China, overall CDG awareness was low in urban and rural areas, but was higher in the former than in the latter (29.1% vs. 19.9%, respectively) [32]. In 10 provinces of China, rural residents had less knowledge, lower usage, and lower awareness of the benefits of nutrition labels than their urban counterparts [33]. Taste, price, health, and convenience are the main driving factors for food choices [34], but people who are driven by health tend to choose healthier foods, while those who do not pay much attention to health may eat convenient snacks [35]. Preventing overweight and obesity among children and adolescents is a public health priority. Comprehensive measures should be taken to improve children and adolescents’ nutritional knowledge, attitudes, and behavior. School years are now regarded as a further window of opportunity when interventions targeting the development or the reversal of becoming overweight or obese are particularly warranted. Eating snacks is a behavior that can be adjusted at the group level to prevent obesity. Therefore, health education that focuses on reducing the intake of snacks in grains and replacing them with more nutritious snacks such as fruits and vegetables may be beneficial. And, in future dietary recommendations and food policies, in addition to nutrients and food groups, food processing may also need to be considered as a food dimension.

This study has several strengths and limitations. The strength of our study was that we found that the snack intake led to a high intake of the food group of cereals, tubers, and beans among children and adolescents with overweight/obesity. This will be of great significance for guiding children to have a reasonable diet. Despite these strengths, there were a number of limitations that need to be acknowledged. Self-reported dietary recalls are subject to measurement errors. The bias caused by other potential confounding factors that did not need to be adjusted in this study, such as seasonal factors, may affect the results. Considering the generalizability of the study results, all of our primary data were from a provincial database. Although there may be regional differences in children’s dietary nutrition, China is currently in a stage of rapid socio-economic transformation and may have similar dietary patterns. However, targeted intervention measures still need to be based on the local nutritional status. There are various factors that affect obesity such as the economic state, education level, smoking, drinking, sleep patterns, and drug, but these variables were not taken into consideration. In future research, these factors will need to be deeply considered.

## 5. Conclusions

Our survey findings show that overweight/obesity children and adolescents consumed significantly more dietary carbohydrates which is associated with the food group of cereals, tubers, and beans than their counterparts without overweight/obesity. The snack intake led to a high intake of the food group of cereals, tubers, and beans among children and adolescents with overweight/obesity, which revealed, as highlighted in our study, the need for guidelines that ensure children and adolescents to not only consume a healthy diet, but especially reduce the intake of snacks including salty soda biscuits, salty moon cakes, sweet cookies, buns, cakes, Dim sum and moon cakes, fried potato chips, French fries, and other fried snacks. Comprehensive measures should be taken to improve children’s nutritional knowledge, establish healthy habits, and develop a lifelong healthy lifestyle.

## Figures and Tables

**Table 1:** Daily nutrient intake among children and adolescents with or without overweight/obesity (ANOVA, *p*-value set at 0.05).

Variables	Obesity (N = 185)		Overweight (N = 255)		Non-Obesity/Overweight (N = 1387)		Total (N = 1827)	
	Mean	SD	Mean	SD	Mean	SD	F	*p*
Protein (g)	64.31	30.59	64.42	29.94	59.02	27.21	2.565	0.078
Fat (g)	69.75	38.91	72.04	29.30	67.79	40.06	0.585	0.557
Carbohydrate (g)	167.15	78.35	140.97	54.98	145.70	71.51	3.464	0.032
Dietary fiber (g)	6.47	5.62	6.17	5.65	5.74	4.97	0.866	0.421
Zn (mg)	8.21	4.40	8.31	4.37	7.71	3.82	1.415	0.244
Fe (mg)	16.32	8.01	15.33	7.81	13.89	6.75	5.232	0.006
Ca (mg)	403.17	254.60	441.82	341.39	386.46	373.78	1.088	0.337
Vitamin C (mg)	50.60	45.92	72.28	234.02	49.14	50.09	2.63	0.073
Vitamin E (mg)	7.09	4.84	8.01	4.82	7.21	5.50	1.07	0.343
Vitamin B1 (mg)	0.74	0.29	0.73	0.31	0.68	0.33	1.49	0.226
Vitamin B2 (mg)	0.72	0.36	0.74	0.44	0.69	0.69	0.349	0.706
Vitamin A (µg RE)	329.06	301.11	383.33	491.21	323.18	513.09	0.671	0.512
Na (mg)	4756.10	3633.29	4316.02	2498.52	3861.39	2177.83	5.619	0.004
K (mg)	1526.25	733.30	1514.81	695.50	1369.07	662.26	3.469	0.032
Mg (mg)	225.70	102.53	225.54	137.05	202.46	88.18	3.934	0.02

**Table 2:** Daily food intake among children and adolescents with or without overweight/obesity (ANOVA, *p*-value set at 0.05).

Food Groups	Obesity (N = 185)		Overweight (N = 255)		Non-Obesity/Overweight (N = 1387)		Total (N = 1827)	
	Mean	SD	Mean	SD	Mean	SD	F	*p*
Cereals, tubers, and beans (g)	245.26	56.79	205.72	67.76	196.61	80.54	7.348	0.001
Snacks (g) *	73.47	64.68	77.24	64.46	52.86	45.50	6.797	0.001
Livestock, poultry, and meat (g)	198.87	163.78	146.32	87.25	103.75	64.47	1.481	0.228
Fish and shrimps (g)	79.64	67.16	58.04	54.53	58.61	45.12	1.54	0.215
Vegetables (g)	192.32	188.63	180.05	70.58	163.49	84.18	2.64	0.072
Fruits (g)	137.17	113.37	69.92	52.17	98.32	111.11	0.82	0.441
Eggs (g)	59.76	23.63	39.15	19.16	42.71	22.61	0.235	0.791
Soybean and nuts (g)	16.13	13.94	16.68	12.04	14.41	13.92	0.082	0.921
Dairy (g)	223.55	87.50	128.37	85.96	193.84	102.29	0.257	0.774
Candied fruit (g)	2.59	1.73	5.05	7.66	5.13	7.64	0.089	0.915
Salt (g)	6.49	3.38	5.61	2.81	5.90	3.25	3.413	0.033
Edible oil (g)	32.06	45.11	28.56	17.97	26.59	19.90	0.143	0.867

* In this analysis, snacks refer to salty soda biscuits, salty moon cakes, sweet cookies, buns, cakes, Dim sum and moon cakes, fried potato chips, French fries, other fried snacks, and snacks were among the food group of cereals, tubers, and beans.

**Table 3:** Ordinal regression of children and adolescents with or without overweight/obesity (Ordinal regression, *p*-value set at 0.05).

Factors	β	Std.E	Wald χ^2^	*p*	95%CI	
					Lower Bound	Upper Bound
Gender	0.885	0.182	23.652	0	0.528	1.242
Age group	0.12	0.266	0.204	0.652	−0.402	0.643
	0.354	0.257	1.9	0.168	−0.149	0.858
	0.182	0.267	0.467	0.495	−0.34	0.704
Cereals, tubers, and beans	−0.724	0.348	4.325	0.038	−1.406	−0.042
	−0.594	0.519	1.309	0.253	−1.61	0.423
Salt	−0.247	0.177	1.948	0.163	−0.594	0.1
Vegetables	−0.042	0.687	0.004	0.951	−1.389	1.305
	−0.016	1.11	0	0.988	−2.191	2.159

**Table 4:** Daily snack intake and its relation to other nutrients among children and adolescents with or without overweight/obesity (ANOVA, *p*-value set at 0.05).

Variables	Obesity (N = 185)		Overweight (N = 255)		Non-Obesity/Overweight (N = 1387)		Total (N = 1827)	
	Mean	SD	Mean	SD	Mean	SD	F	*p*
The energy intake provided by snacks (kcal)	214.62	219.37	220.89	175.02	143.00	134.33	8.305	0.001
The carbohydrate intake provided by snacks (g)	32.42	26.33	35.96	28.97	21.07	22.80	6.316	0.002
Sodium intake provided by snacks (g)	178.69	235.16	216.85	268.46	99.59	116.46	13.955	0.000
The proportion of snacks to the food group of cereals, tubers, and beans	0.25	0.17	0.30	0.20	0.23	0.15	3.692	0.026

## Data Availability

Supporting data can be acquired from the corresponding author.
